# PF-05231023 reduces lipid deposition in apolipoprotein E-deficient mice by inhibiting the expression of lipid synthesis genes

**DOI:** 10.3389/fvets.2024.1429639

**Published:** 2024-07-31

**Authors:** Juan Zhao, Xuelong Liu, Jingyu Yue, Shouquan Zhang, Li Li, Hengxi Wei

**Affiliations:** State Key Laboratory of Swine and Poultry Breeding Industry, National Engineering Research Center for Breeding Swine Industry, Guangdong Provincial Key Lab of Agro-animal Genomics and Molecular Breeding, College of Animal Science, South China Agricultural University, Guangdong, China

**Keywords:** PF-05231023, long-acting FGF21 analogs, lipid metabolism, apolipoprotein E (ApoE), mice

## Abstract

Fibroblast growth factor 21 (FGF21) is a peptide hormone that is primarily expressed and secreted by the liver. The hormone is crucial for regulation of glucose homeostasis, lipid metabolism, and energy balance. Compared with natural FGF21, FGF21 analogs have become drug candidates for the treatment of cardiovascular and metabolic diseases owing to their long half-life and greater stability *in vitro*. Apolipoprotein E (*Apoe)*-knockout (*Apoe*^−/−^) mice exhibit progressive disruptions in lipid metabolism *in vivo* and develop further atherosclerosis pathological features owing to *Apoe* deletion. Therefore, this study used an *Apoe*^−/−^ mouse model to investigate the effects of a long-acting FGF21 analog (PF-05231023) on lipid metabolism and related parameters. Eighteen *Apoe*^−/−^ female mice were fed a Western diet equivalent for 12 weeks, and then randomly assigned to intraperitoneally receive either physiological saline (the control group) or 10 mg/kg PF-05231023 (the treatment group) three times a week for seven consecutive weeks. Body composition, glucose tolerance, blood and liver cholesterol, triglyceride levels, liver vacuolization levels, peri-ovarian white adipocyte hypertrophy, aortic atherosclerotic plaque formation, and the expression of genes related to lipid metabolism in adipose tissue were subsequently assessed before and after treatment. The aortic atherosclerotic plaque area was reduced in mice in the PF-05231023 treatment group compared with that in the saline group. Although the effect of PF-05231023 on the plasma biochemical indexes of mice was small, it significantly reduced lipid levels and lipid droplet accumulation in the liver, and reduced adipocyte hypertrophy in white adipose tissue. Transcriptome analysis of adipose tissue showed that PF-05231023 treatment downregulated the expression of lipid synthesis-related genes and inhibited the sterol regulatory element binding transcription factor 1 gene, thereby improving lipid deposition. PF-05231023 effectively improved the lipid metabolism of *Apoe*^−/−^ mice, demonstrating an anti-atherosclerotic effect and providing a scientific basis and experimental foundation for the clinical treatment of cardiovascular diseases by using long-acting FGF21 analogs.

## 1 Introduction

Excessive consumption of calorie-rich foods leading to obesity-related diseases is now recognized as a major cause of disability around the world (1). Mechanistically, caloric intake that exceeds adipose tissue storage capacity is associated with the accumulation of ectopic lipids in non-adipose organs and the induction of low-grade tissue inflammation, endoplasmic reticulum stress, and insulin resistance (2, 3). These metabolic defects increase the risk of serious diseases, including type 2 diabetes, non-alcoholic steatohepatitis, cardiovascular disease and various forms of cancer. However, to date, specific drugs for these diseases remain limited.

Fibroblast growth factor 21 (FGF21) is a peptide hormone that is primarily expressed and secreted by the liver and adipose tissue (4, 5). The hormone acts in an endocrine manner and targets liver and adipose tissue (6). Increasing evidence suggests an important role for FGF 21 in the regulation of glucose and lipid homeostasis through multifaceted and inter-organ crosstalk. Moreover, FGF 21 has been recognized as a potential target for metabolic abnormalities (7). Although the role of FGF21 in obesity, diabetes, and non-alcoholic fatty liver disease (NAFLD) in humans and animals has been extensively studied (8–10), the hormone's short circulating half-life (30–120 min) and its tendency to aggregate *in vitro* limit its clinical application (11). To overcome these limitations, the long-acting FGF21 analog, PF-05231023, was developed. The analog possesses better anti-aggregation and *in vivo* degradation effects than that of natural FGF21 (12–14). PF-05231023 contains two modified human FGF21 molecules that are linked to the humanized immunoglobulin 1 antibody backbone, which was designed to prolong the analog's half-life and bioavailability (15). Compared with that of natural FGF21, PF-05231023 exhibits a 70-fold increase in half-life, demonstrating promising clinical potential *in vitro*. Similarly, administration of LY2405319, an FGF21 analog, reduced atherosclerotic plaques and blood lipid levels in *Apoe*^−/−^ mice (16). Clinical trials have demonstrated the potential of recombinant FGF21 for the treatment of type 2 diabetes, obesity, and other comorbidities, but its physiological role in atherosclerosis (AS) has only gained attention in recent years (16, 17).

In studies involving apolipoprotein E-knockout (*Apoe*^−/−^) mice, treatment with recombinant human FGF21 prevented the formation of atherosclerotic plaques (18).

However, the specific mechanism by which PF-05231023 modulates lipid metabolism and protects against AS in *Apoe*^−/−^ mice remains unclear, and its impact on lipid metabolism-related pathways has yet to be elucidated. Therefore, we hypothesized that PF-05231023 would improve lipid metabolism and atherogenesis in *Apoe*^−/−^ mice. Hence, in this study, we used an *Apoe*^−/−^ mouse model of lipid metabolism disorder, using mice fed a Western diet equivalent (WD), to investigate the specific mechanism by which PF-05231023 alleviates lipid metabolism disorders. We investigated this from a novel perspective of adipose tissue, with the expectation of providing insights for the development of more effective and safer drugs for the treatment of metabolic diseases.

## 2 Materials and methods

### 2.1 Experimental reagents

PF-05231023 (AbMole Company; M10048, Shanghai, China), a total cholesterol (TC) test kit and triglyceride (TG) test kit (Nanjing Jiancheng Biotechnology Research Institute; A111-1-1, A110-1-1, Nanjing, China), D-anhydrous glucose (Solarbio; G8150, Beijing, China), hematoxylin and eosin (HE) staining solutions (Reagan Biotech; DH001, DH0044, Beijing, China), Oil Red O dye solution (Wuhan Seville Biotechnology Co., Ltd.; G1015, Wuhan, China), and TRIzol reagent (Takara; T9108, Beijing, China) were used in this study. The reverse transcription kit and quantitative polymerase chain reaction (PCR) detection kit were both procured from Vazyme (R323-01, Q711-02, Nanjing, China).

### 2.2 Animal experiments

Eighteen 4-week-old female *Apoe*^−/−^ C57BL/6 mice, weighing 17–20 g, were purchased from the Shanghai Model Organisms Center, Inc. (Shanghai, China). The animal quality was certified as SYXK (Shanghai, China) 2019-0002. Six 4-week-old C57BL/6 female mice with the same genetic background, weighing 16–17 g, were procured from the Guangdong Medical Experimental Animal Center with an animal quality license of SYXK (Guangdong, China) 2022-0002. All animal experiments strictly complied with the regulations of the Animal Ethics Committee of South China Agricultural University. The mice were housed and fed at room temperature (22 ± 2°C), with a 12-h light/12-h dark cycle and had free access to food and drinking water.

### 2.3 Experimental design

After acclimating to the environment for 1 week, the mice were randomly divided into two groups: *Apoe*^−/−^ + WD + PF-05231023 (*n* = 6) and *Apoe*^−/−^ + WD + saline group (*n* = 6). These groups were fed a WD for 12 weeks to build the AS model, followed by a 7-week treatment period of either 10 mg/kg of PF-05231023 (15) or an equivalent volume of saline intraperitoneally injected on Monday, Wednesday, and Friday every week. Additionally, C57BL/6 female mice + control diet (WT + Control) served as the healthy control group (*n* = 6), and *Apoe*^−/−^ mice + control diet (*Apoe*^−/−^ + Control) served as the transgenic control group (*n* = 6). The composition of the WD and control diet is shown in Table 1.

**Table 1:** Western diet equivalent (WD) and control diet compositions.

**Nutrient**	**WD (purified) feed**		**WD (control) feed**	
	**Mass ratio %**	**Energy ratio %**	**Mass ratio %**	**Energy ratio %**
Protein	17.5	15.5	17	17
Carbohydrate	48.5	42.5	71	73
Fat	21	42	4.3	10
Total	87	100	92.3	100
Energy	4.7 kcal/g		3.9 kcal/g	
Product	Mass ratio g/kg	kcal	Mass ratio g/kg	kcal
Casein	195	780	195	780
DL-Methionine	3	12	3	12
Sucrose	341	1,364	341	1,364
Com starch	150	600	504.4	2,017.6
Cellulose	50	0	50	0
Natural cream	210	1,890	52.5	472.5
Ethoxyquin	0.04	0	0.04	0
Mineral mix AIN76A	35	16.5	35	16.5
Calcium carbonate	4	0	4	0
Vitamin Mix AIN-76A	10	39	10	39
Choline bitartrate	2	0	2	0
Cholesterol	1.5	0		
TOTAL	1,001.54	4,701.5	1,196.94	4,701.6

**Figure 1:**
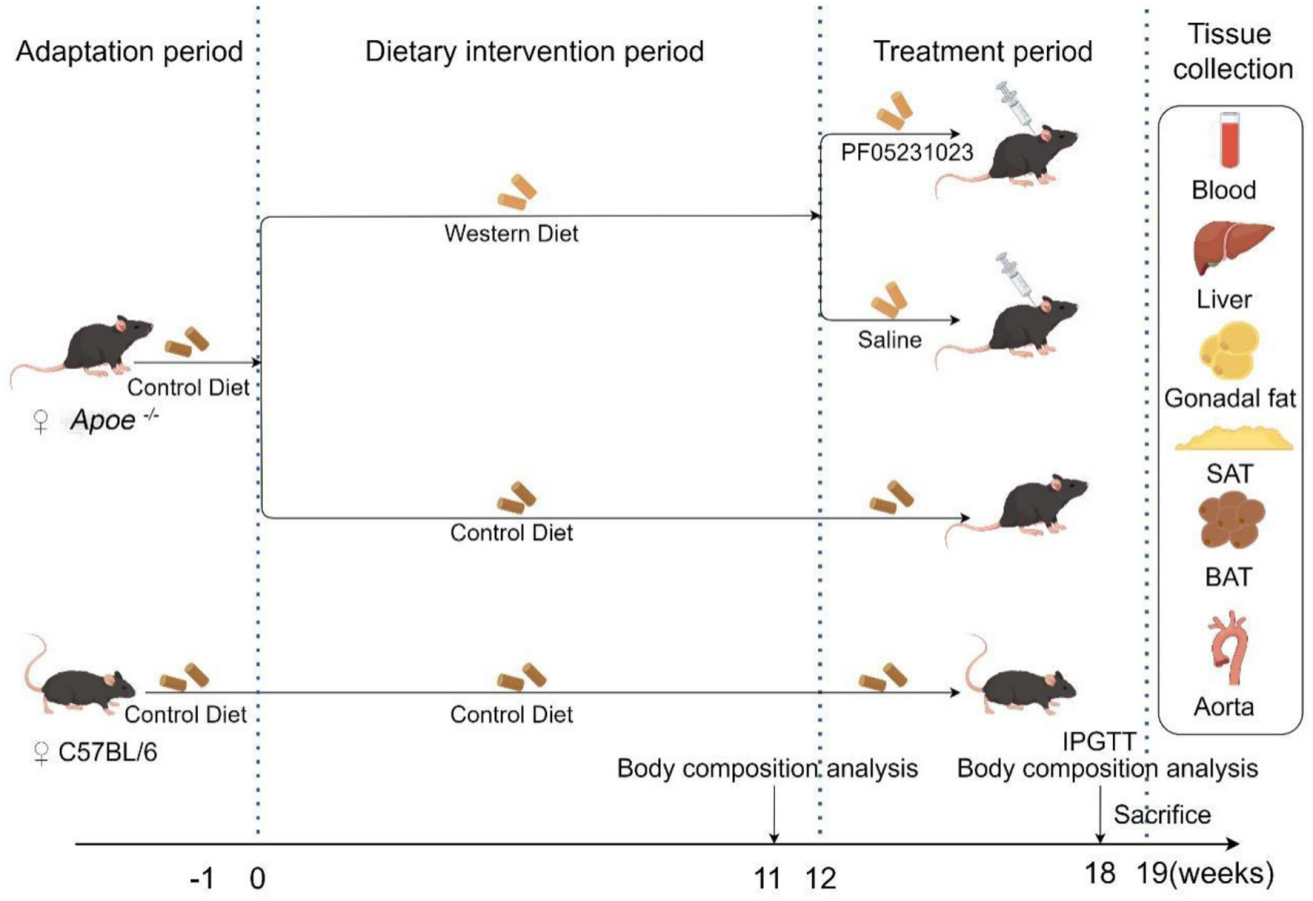
Experimental flowchart (created using Figdraw). *Apoe*^−/−^, Apolipoprotein E knockout; IPGTT, intraperitoneal glucose tolerance test; SAT, subcutaneous adipose tissue; BAT, brown adipose tissue.

Throughout the feeding period, the weights of the mice were recorded weekly. Their food and water were also replaced weekly. Body composition analysis of the mice was conducted at weeks 12 and 19 of feeding, and an intraperitoneal glucose tolerance test (IPGTT) was performed during the 7th week of treatment. Blood samples were collected from the eyeballs and perfused with physiological saline to dissect the aorta. Subsequently, the mouse liver, subcutaneous fat, brown fat, and white fat around the ovaries were stored at −80 °C for subsequent analysis. Experimental design is shown in Figure 1.

### 2.4 Body composition analysis

Body composition analysis was performed on mice at weeks 12 and 19. Before testing, place the mice between two paper cups, gently squeeze them, and perform stress induced bowel movements to minimize experimental errors. Subsequently, they were placed in a live animal body composition analyzer to record their body weight, body fat weight, lean meat weight, and their percentage.

### 2.5 IPGTT

Mice were transferred to a clean cage, where they were fasted for 5 h, during which they had access to water. After the fasting period, their weight was measured, and the appropriate amount of glucose injection was calculated based on their weight. Fasting blood glucose levels were measured, and a glucose solution was injected into the abdominal cavity at a standard concentration of 2 mg/g (19). Blood glucose levels were monitored at 15, 30, 60, 90, and 120 min post-injection using a blood glucose meter.

### 2.6 Organize sample collection

Blood collection from the eyeballs: After anesthetizing the mice with ether (20), the mice were fixed with one hand, and the eyeballs were removed with sterilized forceps; the blood was collected into 1.5 ml tubes, and left to stand for 4 h. The blood was centrifuged at 3,000 rpm for 15 min at 4°C in a centrifuge (Eppendorf, Hamburg, Germany), and the supernatant was pipetted into 200 μl tubes and store it in a −80°C freezer for further research.

After completion of blood sampling, the mice were executed by dislocation method, and the lower limbs and head were fixed on a foam plate to take white adipose tissue around the ovaries (sexual fat), subcutaneous fat, and brown fat of mice. One side was fixed in paraformaldehyde for subsequent embedding; the other side was placed in liquid, the other side was placed in liquid nitrogen and stored in the refrigerator at −80°C.

Then, the thoracic cavity of the mice was cut open from the diaphragm upward to expose the heart, and a small opening was cut out of the right apical heart, and pre-cooled saline was injected from the left apical heart with a 1 ml syringe until the outflow from the opening of the right apical heart was clear and the color of the liver was gradually changed to lighter; the aorta of the mice was dissected under the body microscope, and the peri-arterial blood vessels and fat were stripped off, and the aortic arch was fixed in paraformaldehyde for subsequent staining analysis (21).

### 2.7 Biochemical analysis of serum and liver

The collected blood samples were centrifuged at 3,000 rpm for 15 min at 4°C to separate the serum. Liver tissue (30 mg) was homogenized with phosphate-buffered saline, and the supernatant was collected for further analysis. Serum and liver TC, and TG levels were measured according to the manufacturer's instructions provided with the reagent kit.

### 2.8 Histological analysis of liver, aorta, and adipose tissue

White fat surrounding the left hepatic lobule, aorta, and left ovary was fixed in 4% paraformaldehyde for 24 h. Subsequently, the liver and fat were embedded in paraffin sections (thickness = 5 μm) and subjected to HE staining. Lipid accumulation in the liver and adipocyte hypertrophy in the fat were observed and photographed under a 20 × biological microscope (SOPTOP, EX20, Suzhou, China). The aorta was longitudinally placed on a black gel, and blood vessels were cut longitudinally under a stereomicroscope (Olympus Corporation, SZ2-ILST, Tokyo, Japan). Oil Red O staining was performed, and lipid deposition in the aorta was observed using a 4 × microscope (SOPTOP, EX20). Image View software was used for image stitching, and analysis was performed using ImageJ1 software.

### 2.9 Extraction of RNA from mouse adipose tissue

Three mice were randomly selected from the *Apoe*^−/−^ + WD + PF-05231023 and *Apoe*^−/−^ + WD + saline group (*n* = 6). The white fat tissue surrounding the ovaries was collected, and total RNA was extracted from this adipose tissue using TRIzol reagent. The purity of samples was assessed using a NanoPhotometer^®^ Thermo Fisher spectrophotometer (Massachusetts, USA), whereas the integrity and concentration of the RNA samples were determined using the Agilent Technologies (CA, USA) 2100 RNA Nano 6000 Assay Kit.

### 2.10 Library construction and sequencing

Following total RNA extraction, eukaryotic mRNA was enriched using oligo (dT) magnetic beads. A buffer was then added for mRNA fragmentation, and mRNA was used as a template to synthesize the complementary DNA (cDNA) first and second strands. The cDNA was purified, and the resulting purified double-stranded cDNA was subjected to end repair, addition of base A, and addition of sequencing adapters. Fragments were screened to recover ~350 bp of cDNA, and PCR enrichment was performed to obtain a cDNA library. After passing the quality inspection using Qubit 3.0 and Agilent 2100, the constructed library was sequenced using a high-throughput sequencing platform (Agilent Technologies Inc, California, USA) with a PE150 sequencing strategy. The sequencing was conducted by Annoroad Gene Technology (Beijing, China).

### 2.11 Differential gene analysis and functional enrichment

Gene differential expression analysis was performed using DESeq2, with screening criteria for differential genes set at FoldChange ≥1.5, *p*-value ≤ 0.05, and *p*adj ≤ 1. For Gene Ontology (GO) and Kyoto Encyclopedia of Genes and Genomes (KEGG) enrichment analysis of differentially expressed mRNA, we used the Database for Annotation, Visualization, and Integrated Discovery (https://david.ncifcrf.gov/tools.jsp). We used the microbiome websites (http://www.bioinformatics.com.cn and https://www.omicshare.com/tools/) for analysis and visualization.

### 2.12 Real-time fluorescence quantitative reverse transcription PCR

To validate RNA sequencing (RNA-Seq) data, eight differentially expressed genes (DEG) related to metabolism were randomly selected for quantitative reverse transcription PCR (qRT-PCR) validation, with β-actin serving as an internal reference gene. Primer sequences and related information are provided in Table 2. The RNA sample used for qRT-PCR amplification was the same as the RNA sample used for constructing the RNA-Seq library mentioned above when using the Vazyme reverse transcription kit.

**Table 2:** Study primer sequences.

**Gene**	**Accession number**	**(5^′^-3^′^)**	**(3^′^-5^′^)**
β-actin	NM_007393	GTGACGTTGACATCCGTAAAGA	GCCGGACTCATCGTACTCC
*Agpat2*	NM_026212.2	CAGCCAGGTTCTACGCCAAG	TGATGCTCATGTTATCCACGGT
*Angptl8*	NM_001080940.1	CCCTCAATGGCGTGTACAGA	CCACCTGAATCTCCGACAGG
*Mrap*	NM_029844.4	CCTGGCTACCTTCGTGGTG	GGGAGGTTGAAGCTGTGAGTC
*Pla2g2e*	NM_012044.2	CCAGTGGACGAGACGGATTG	AGCAGCTCTCTTGTCACACTC
*Dbi*	NM_007830.4	CAAGCTACTGTGGGCGATGTA	CACATAGGTCTTCATGGCACTTT
*B3gnt5*	NM_001159407.1	TGGCTTTGTAAACAATTCCCTGT	GAACGTCGGCCATAGTTTTCA
*Cr2*	NM_001368765.1	TGTCATCCCGACATGCAAAGA	ACACTGACTAGAGGGTTGTCC
*Ltf*	NM_008522.3	TGATGACACCCGGAAACCTG	TCCCAAACTGAACCTGTTGGT
*Ucp2*	NM_001417452.1	GTGGTGGTCGGAGATACCAGA	GGGCAACATTGGGAGAAGTCC

### 2.13 Statistical analysis

Unless otherwise specified, statistical analysis was conducted using one-way analysis of variance (ANOVA). We performed multiple comparisons after one-way ANOVA, compared each column, and rated the average of each column. Data is expressed as mean ± standard error mean, and *p* < 0.05 is considered statistically significant. All statistical analyses were performed using GraphPad Prism 8.0.

## 3 Results

### 3.1 The effects of PF-05231023 administration on body weight and fat in Apoe ^−*/−*^ mice

**Figure 2:**
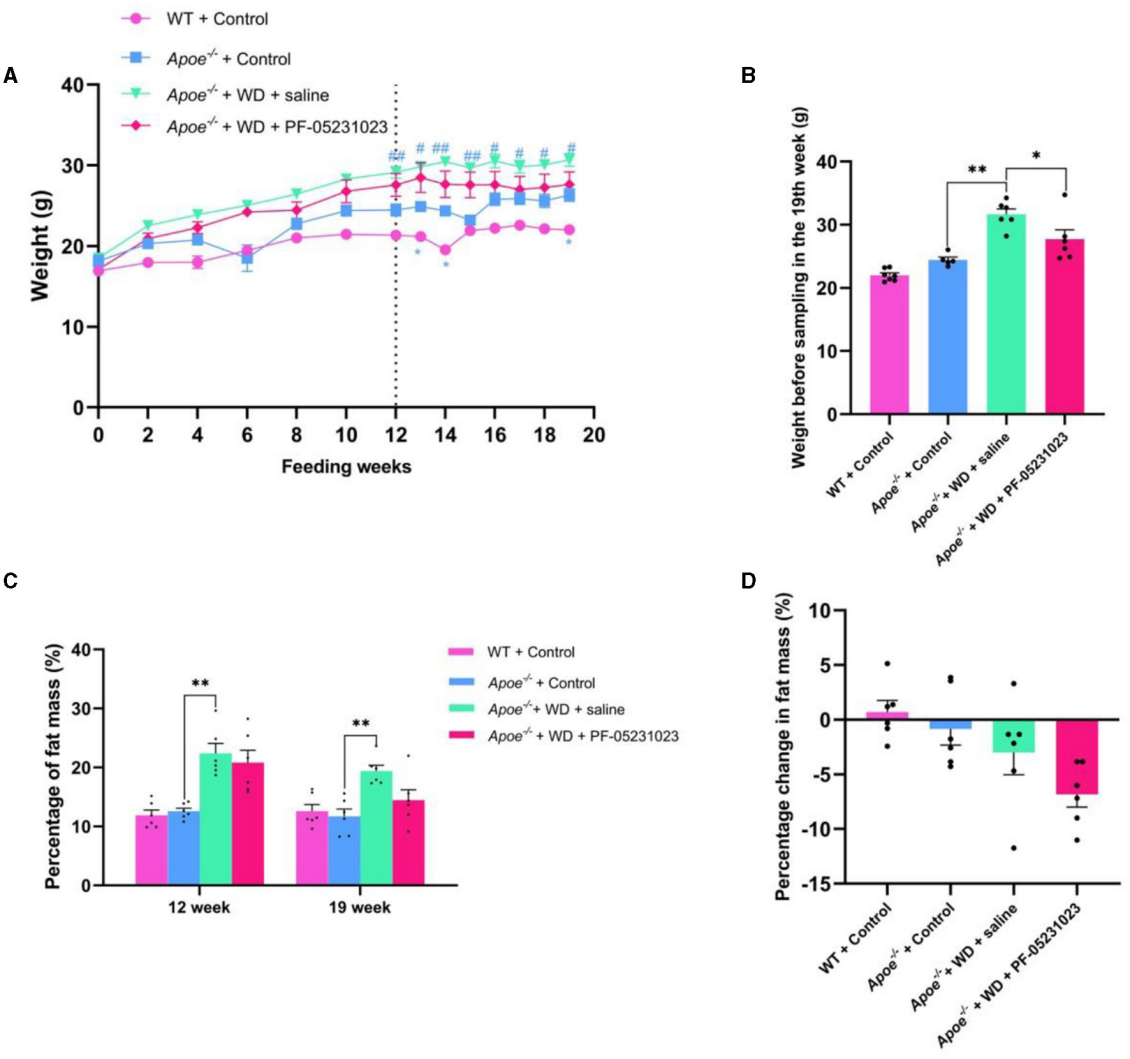
**(A)** Changes in body weight of *Apoe*^−/−^ mice during feeding and treatment. *indicates a significant difference between the WT + Control group and the *Apoe*^−/−^ + Control group (*p < 0.05, **p < 0.01), whereas ^#^ indicates a significant difference between the *Apoe*^−/−^ + Control group and the *Apoe*^−/−^ + WD + saline group (^#^*p* < 0.05, ^##^*p* < 0.01). Control represents the Western-style diet control group, and WD indicates the Western-style diet purification group. **(B)** Body weight of mice before sampling. **(C)** Fat content before and after treatment. **(D)** Fat mass change after treatment.

During the first week of feeding, there was no significant difference in body weights among all mice, although the *Apoe*^−/−^ mice exhibited a slightly higher body weight than that of the WT + Control group. After 12 weeks of the feeding regimen, there was no significant difference in weights between the *Apoe*^−/−^ + WD + PF-05231023 group and the *Apoe*^−/−^ + WD + saline group. However, a significant difference in weights was noted between the WT + Control group and the *Apoe*^−/−^ + Control group (*p* < 0.05). PF-05231023 treatment was initiated after the 12th week. During the 7-week treatment period, the weight of mice in the *Apoe*^−/−^ + WD + PF-05231023 group increased in the first week, gradually decreased, and then remained stable thereafter. The weight of the *Apoe*^−/−^ + WD + saline group showed a slight upward trend; however, the difference was not significant between the two groups. Furthermore, a significant difference in body weight was noted between the *Apoe*^−/−^ + Control group and the *Apoe*^−/−^ + WD + saline group (*p* < 0.01). A significant difference in body weight was also observed between the WT + Control group and the *Apoe*^−/−^ + Control group (*p* < 0.05, Figure 2A), as well as between the *Apoe*^−/−^ + WD + PF-05231023 group and the *Apoe*^−/−^ + WD + saline group before mouse sampling (*p* < 0.05, Figure 2B). These results indicate that PF-05231023 treatment affected the body weight of *Apoe*^−/−^ mice.

Body composition analysis conducted before and after treatment revealed no significant fluctuation in fat content between the WT + Control and *Apoe*^−/−^ + Control groups (Figure 2C). Both the *Apoe*^−/−^ + WD + PF-05231023 and *Apoe*^−/−^ + WD + saline groups showed a decrease in fat content before and after treatment (Figure 2C). Specifically, the saline group showed a decrease of 2.99%, whereas the PF-05231023 group exhibited a decrease of 6.82%. The proportion of decrease in the PF-05231023 group was more than twice that in the control group. Overall, the proportion of fat that decreased in the PF-05231023 group was higher than that in the saline group (Figure 2D). These results indicate that PF-05231023 causes s a slight decrease in fat content in *Apoe*^−/−^ mice.

### 3.2 The effects of PF-05231023 on blood glucose levels in Apoe*^−/−^* mice

**Figure 3:**
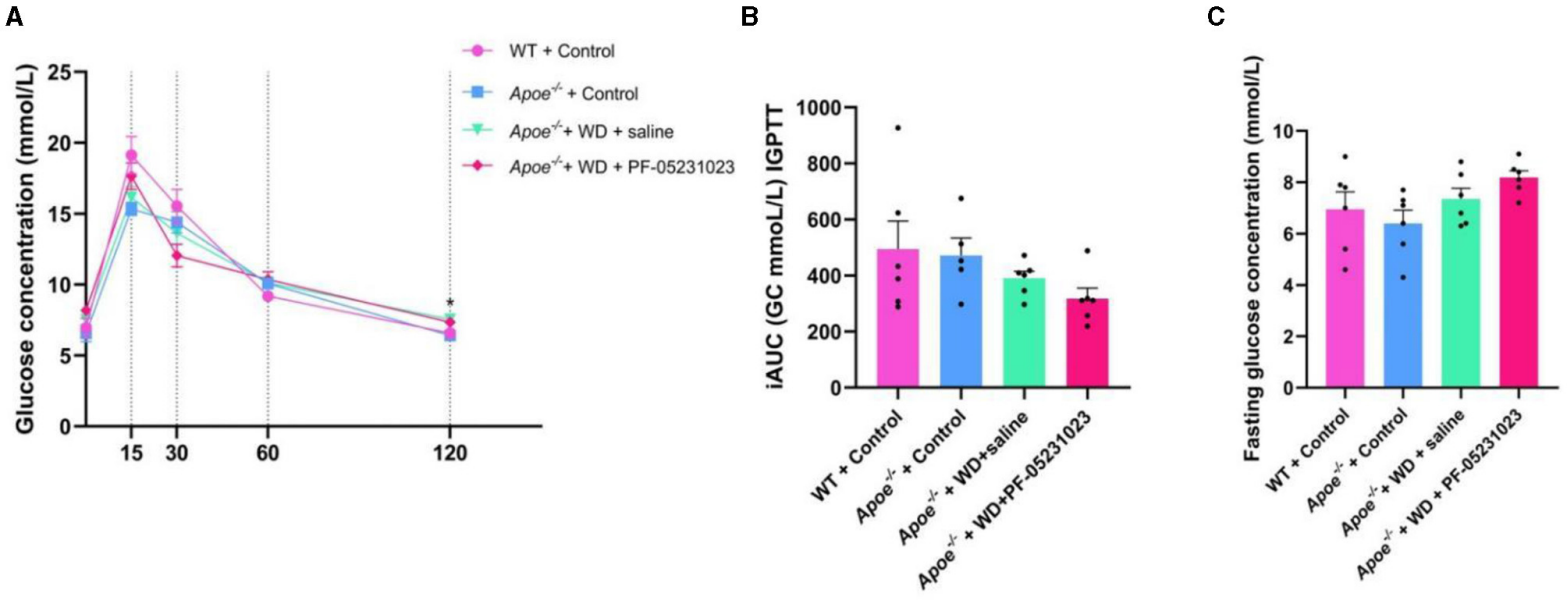
**(A)** Detection of glucose levels within 0–120 min of the intraperitoneal glucose tolerance test (IPGTT) in mice at the end of treatment, *indicates a significant difference between the WT + Control group and the *Apoe*^−/−^ + Control group **(B)** Increment of area under the curve (iAUC) of IPGTT across the four groups. **(C)** Fasting blood glucose concentration in different groups of mice after 7 weeks of treatment. GC, glucose.

At the end of 7 weeks of treatment, mice were subjected to an IPGTT. Within 120 min of the IPGTT, mice treated with PF-05231023 showed the fastest decrease in blood glucose levels at 30 min compared with that in the group injected with physiological saline (Figure 3A). The area under the glucose curve was also lower than that for the control group (Figure 3B). Although blood glucose levels were slightly higher in the fasting state (Figure 3C), no statistical difference was observed. These results indicate that the effect of PF-05231023 treatment on blood glucose levels in *Apoe*^−/−^ mice was modest. Plasma lipid level analysis revealed no significant decrease in TC and TG levels in the plasma of mice treated with PF-05231023 compared with that of mice treated with physiological saline (data not shown).

### 3.3 The effects of PF-05231023 on the aorta of Apoe*^−/−^* mice

**Figure 4:**
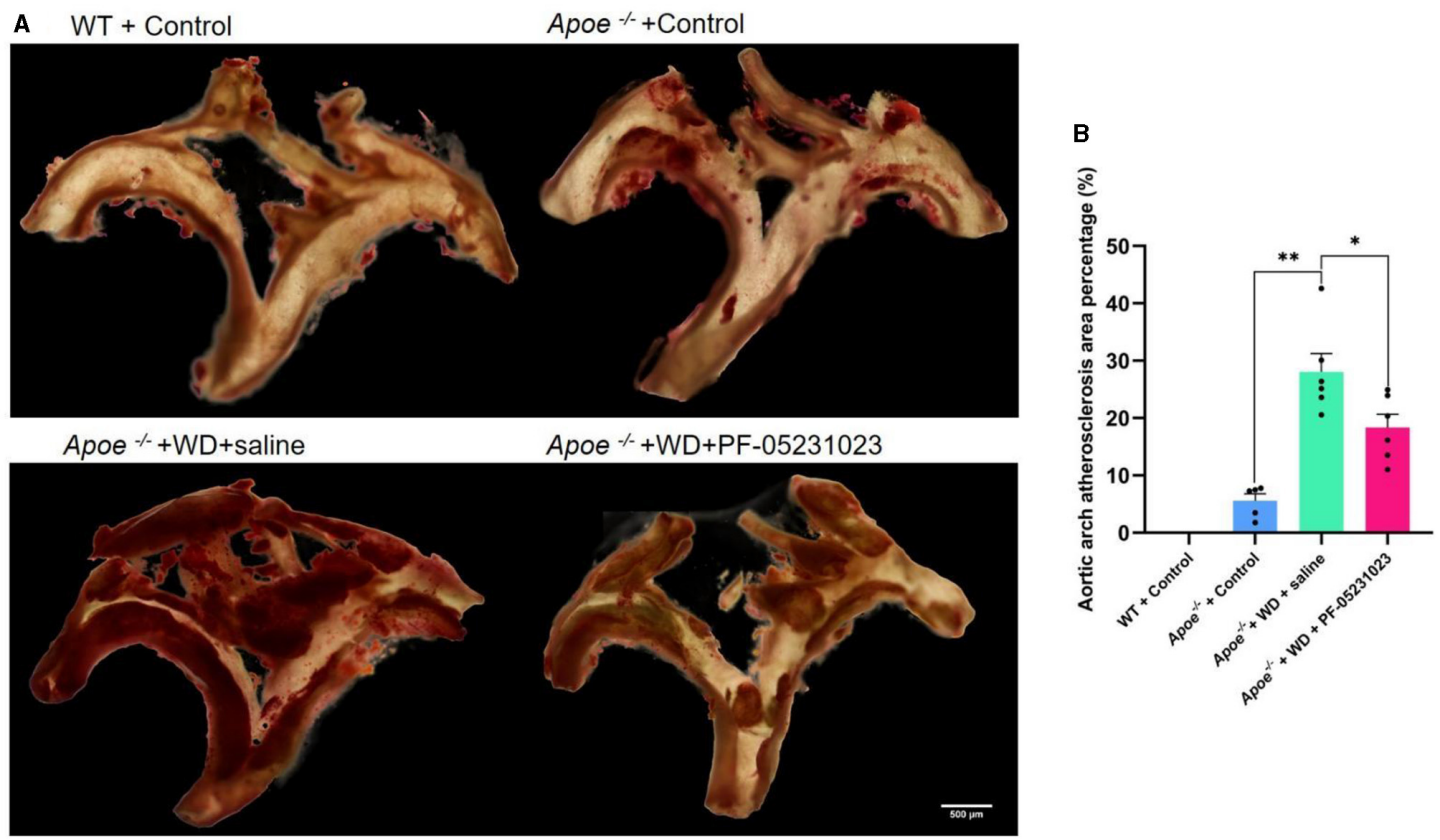
**(A)** Representative images of Oil Red O-stained aortic arch from the four mouse groups. Red stain indicates plaque accumulation. **(B)** Atherosclerotic plaque area after treatment. Scale bar: 500 μm. *means there is a significant difference between the two groups (*p* < 0.05), **means there is a highly significant difference between the two groups (*p* < 0.01).

Oil Red O staining analysis was performed on the aortic arch. The WT + Control group exhibited a normal aortic wall with no lipid deposition. However, partial lipid deposition was observed in the aortic wall of the *Apoe*^−/−^ + Control group. The intima of the aorta in the *Apoe*^−/−^ + WD + saline group appeared relatively fragile, with a substantial amount of lipid deposition noted on the vessel wall. Compared with that in the saline injection group, the *Apoe*^−/−^ + WD + PF-05231023 group showed reduced lipid deposition on the vessel wall and milder plaque lesions (Figure 4A). Furthermore, the plaque area was significantly smaller compared with that in the saline injection group (Figure 4B; *p* < 0.05). These results indicate that PF-05231023 reduced plaque accumulation in the aortic arch of *Apoe*^−/−^ mice.

### 3.4 The effects of PF-05231023 on lipid deposition and biochemical levels in the liver of Apoe*^−/−^* mice

**Figure 5:**
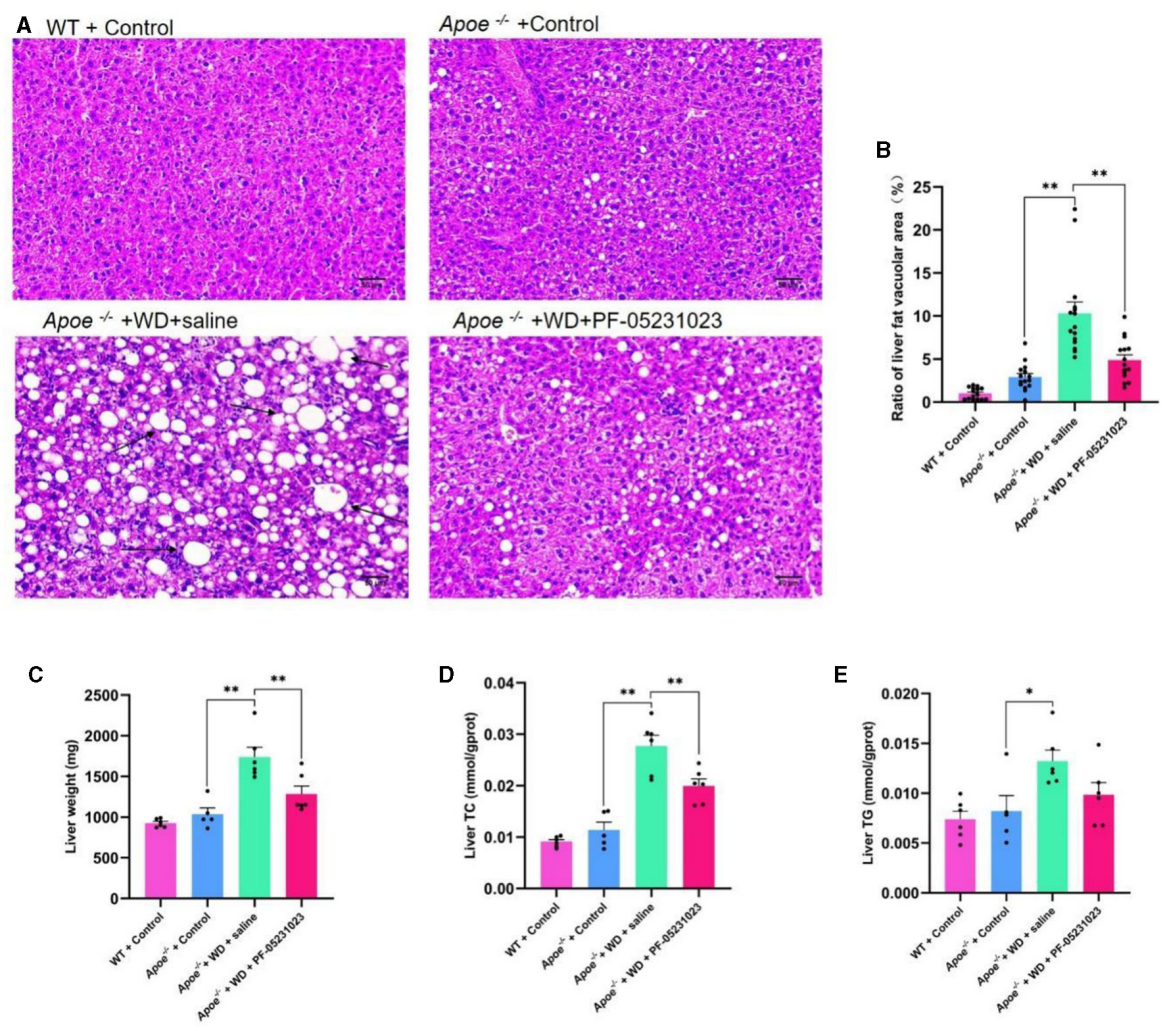
**(A)** Representative images of hematoxylin and eosin (HE)-stained livers from the four mouse groups. The black arrows indicates the main lesions. **(B)** Percentage of liver vacuolization. **(C)** Liver weight after sampling. **(D)** Total cholesterol (TC) content in the liver. **(E)** Liver triglyceride (TG) levels. Scale bar: 50 μm. *means there is a significant difference between the two groups (*p* < 0.05), **means there is a highly significant difference between the two groups (*p* < 0.01).

Liver sections stained with HE were examined under a microscope at 200 × magnification. Compared with that in the group injected with saline, mice treated with PF-05231023 exhibited a smaller area of hepatic fat vacuolization (Figure 5A) and a significant decrease in lipid deposition (Figure 5B, *p* < 0.01). This decrease approached the level observed in the *Apoe*^−/−^ + Control group, indicating that PF-05231023 improved hepatic steatosis in *Apoe*^−/−^ mice. In addition, the TC levels in the livers of mice from the *Apoe*^−/−^ + WD + PF-05231023 group were lower than those in the saline group (Figure 5D). Although TG levels did not show statistical differences, there was a slight trend of decrease (Figure 5E). PF-05231023 also improved liver weight (Figure 5C).

### 3.5 The effects of PF-05231023 on Apoe*^−/−^* mouse adipose tissue

**Figure 6:**
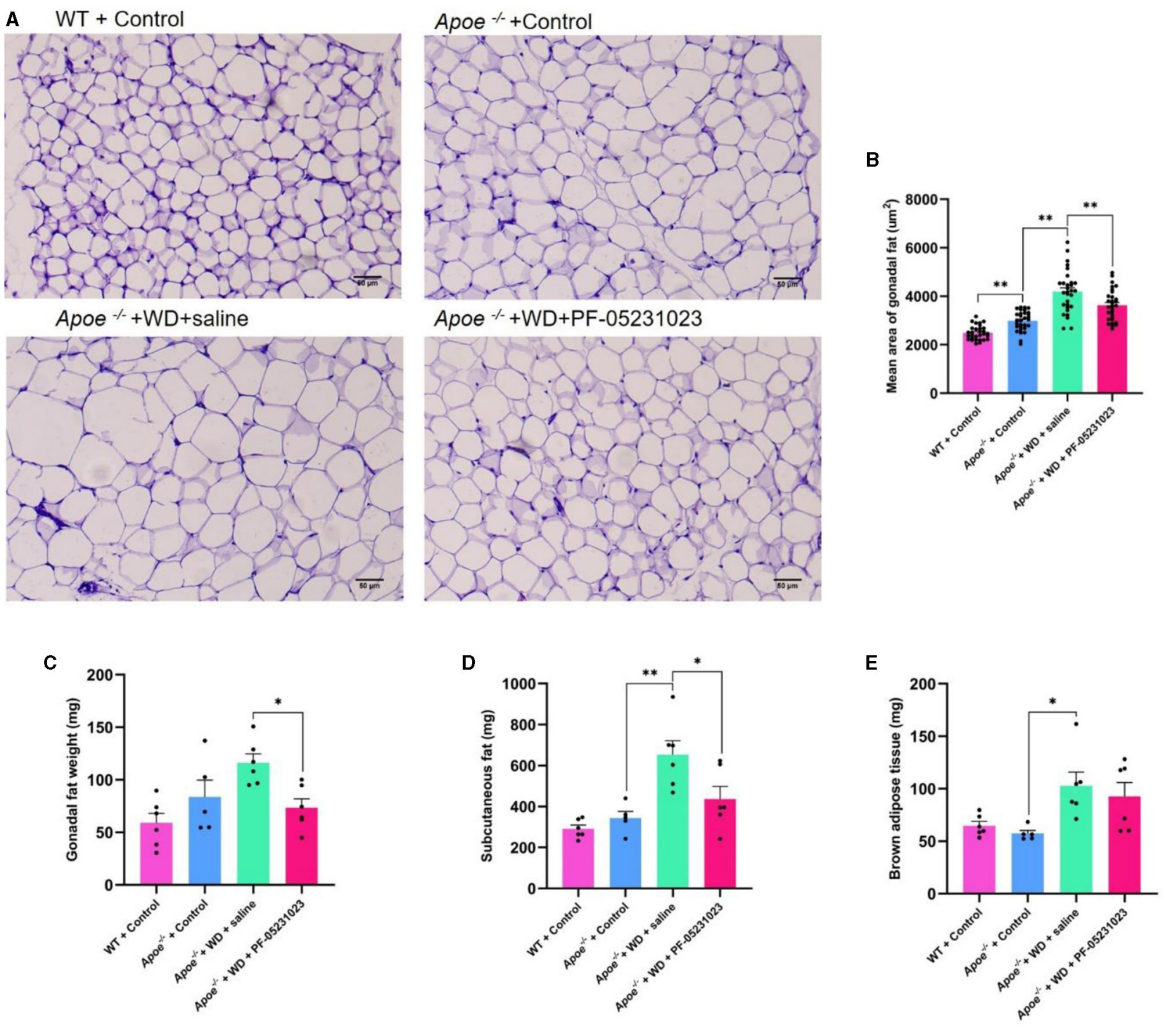
**(A)** Representative images of Hematoxylin and eosin (HE)-stained white adipose tissue around the ovaries of mice in the four study groups. **(B)** Average area of white adipocytes around the ovaries. **(C)** Weight of white fat around the ovaries after sampling. **(D)** Subcutaneous fat weight after sampling. **(E)** Weight of brown fat after sampling. Scale bar: 50 μm. *means there is a significant difference between the two groups (*p* < 0.05), **means there is a highly significant difference between the two groups (*p* < 0.01).

Sections of white adipose tissue around the ovaries were subjected to HE staining and examined using a microscope at 200 × magnification. The adipocytes in the WT + Control group mice were tightly arranged, had small cell diameters, and possessed small volumes (Figure 6A). Compared with those in the WT + Control group, the *Apoe*^−/−^ + Control group exhibited slightly larger cell diameters and volumes (Figure 6A). The volume and diameter of adipocytes in the *Apoe*^−/−^ + WD + saline group were increased, exhibiting characteristics of uneven cell size and irregular shape (Figure 6A). However, in the *Apoe*^−/−^ + WD + PF-05231023 group, adipocytes were smaller than those in the *Apoe*^−/−^ + WD + saline group, with reduced volume and diameter and more regular arrangement (Figure 6A). The average area of white adipocytes around the ovaries significantly decreased in the average area of adipose tissue cells in the *Apoe*^−/−^ + WD + PF-05231023 group compared with that in the *Apoe*^−/−^ + WD + saline group (Figure 6B, *p* < 0. 05). Weight statistics were obtained for white, subcutaneous, and brown fat around the ovaries after sampling. Compared with that in the *Apoe*^−/−^ + WD + saline group, the *Apoe*^−/−^ + WD + PF-05231023 group showed a significant decrease in the weight of white fat around the ovaries (Figure 6C) and subcutaneous fat (Figure 6D, *p* < 0.05), whereas no statistically significant difference was noted in terms of brown fat (Figure 6E, *p* > 0.05).

### 3.6 DEGs and functional annotation analysis

**Figure 7:**
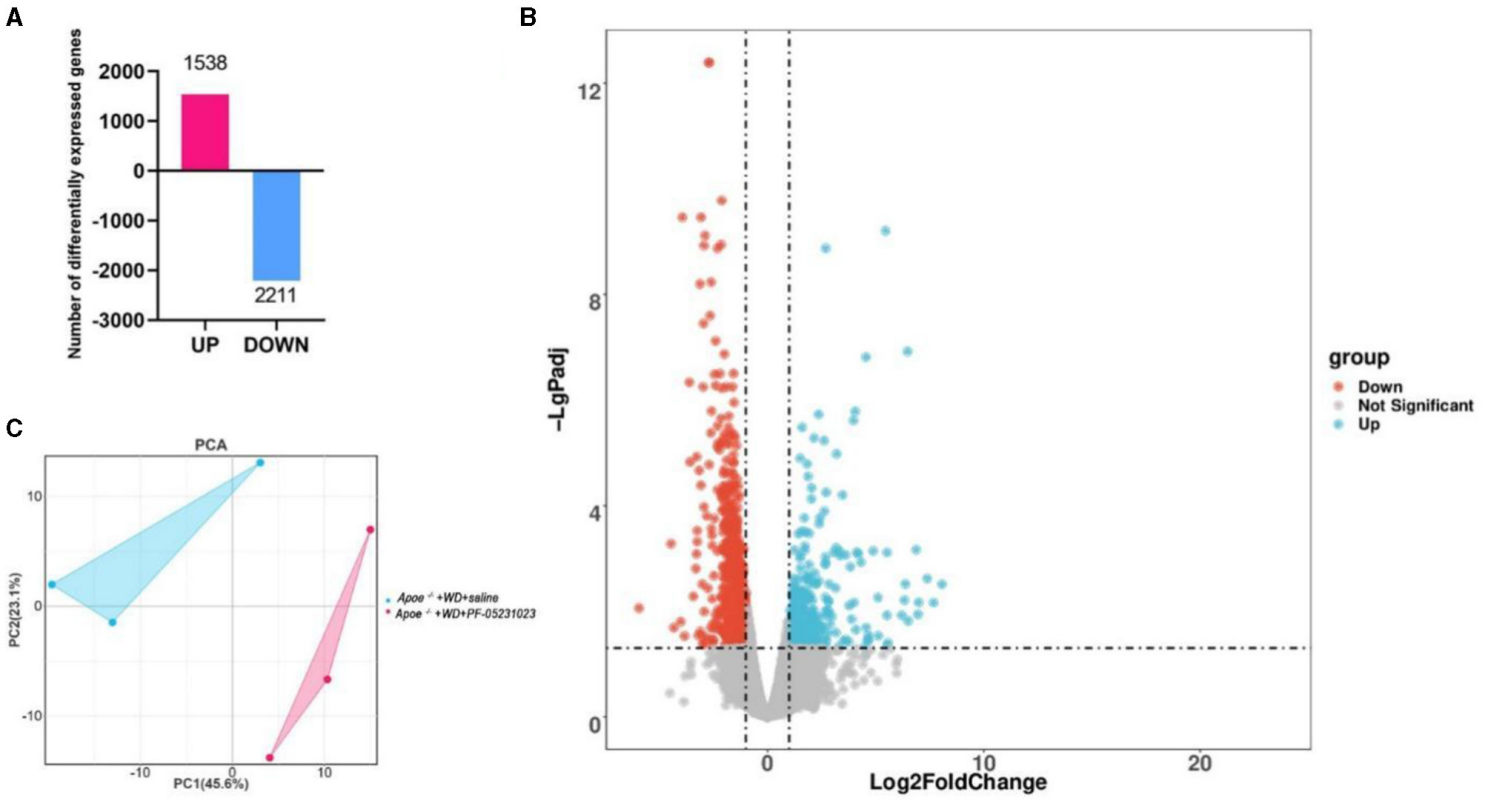
DEG profiles of white adipose tissue around the ovaries of *Apoe*^−/−^+ WD + PF-05231023 and *Apoe*^−/−^ + WD + saline mice. **(A)** The number of DEGs between the *Apoe*^−/−^+ WD + PF-05231023 and *Apoe*^−/−^+ WD + saline groups. **(B)** Volcano map of DEGs between the *Apoe*^−/−^+ WD + PF-05231023 and *Apoe*^−/−^+ WD + saline groups. Red represents significant upregulation, blue represents significant downregulation, and gray represents no significant difference. **(C)** Principal component (PC) analysis was performed on the top 400 significantly different gene expression levels between the *Apoe*^−/−^+ WD + PF-05231023 and *Apoe*^−/−^+ WD + saline groups.

We conducted sequencing analysis of the white adipose tissue around the ovaries in the *Apoe*^−/−^ + WD + PF-05231023 group and the *Apoe*^−/−^ + WD + saline group. Three biological replicates were used for each group. Our sequencing results revealed a total of 3,749 DEGs between *Apoe*^−/−^ + WD + PF-05231023 and *Apoe*^−/−^ + WD + saline mice, including 1,538 upregulated genes and 2,211 downregulated genes (Figure 7A; screening parameter | log_2_ Fold change | ≥ 1 and *q* < 0.05). The volcano plot analysis a more intuitive up-and-down regulation of DEGs (Figure 7B). Principal component analysis of these DEGs demonstrated high similarity within the groups (Figure 7C).

**Figure 8:**
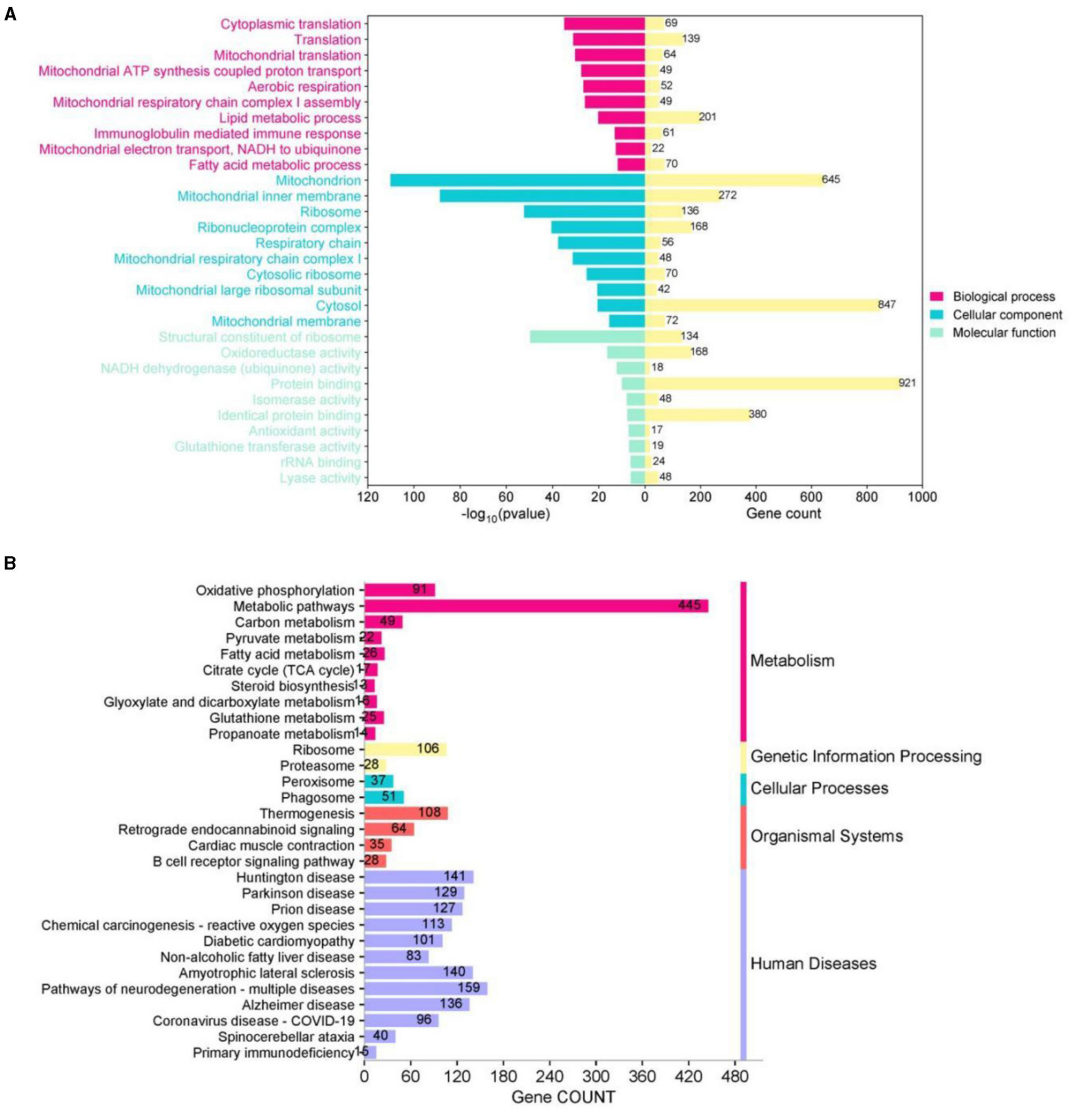
Functional classification of DEGbetween *Apoe*^−/−^+ WD + PF-05231023 and *Apoe*^−/−^+ WD + saline groups. **(A)** Gene ontology (GO) and **(B)** Kyoto Encyclopedia of Genes and Genomes (KEGG) functional enrichment analysis of DEGs.

We used the GO and KEGG databases to classify DEG functions (Figure 8). In the top 10 biological processes (BP), cellular component (CC), and molecular function (MF), GO enrichment analysis showed that BP was primarily associated with translation, aerobic respiration, lipid metabolism, and immune response processes, whereas CC was mainly concentrated in mitochondria, ribosomes, respiratory chains, and other cellular parts. In MF, enrichment was observed in ribosomal structural components, oxidoreductase activity, and homologous protein binding, and other functional aspects (Figure 8A). We also annotated DEGs based on the KEGG database and noted that 3,749 DEGs were concentrated in 81 KEGG pathways within six categories: metabolism, genetic information processing, environmental information processing, cellular processes, biological systems, and human diseases. Among these pathways, apart from human diseases, the most DEGs were in metabolic processes, including oxidative phosphorylation, metabolic pathways, fatty acid metabolism, steroid biosynthesis, glutathione metabolism, and propionic acid metabolism (Figure 8B).

**Figure 9:**
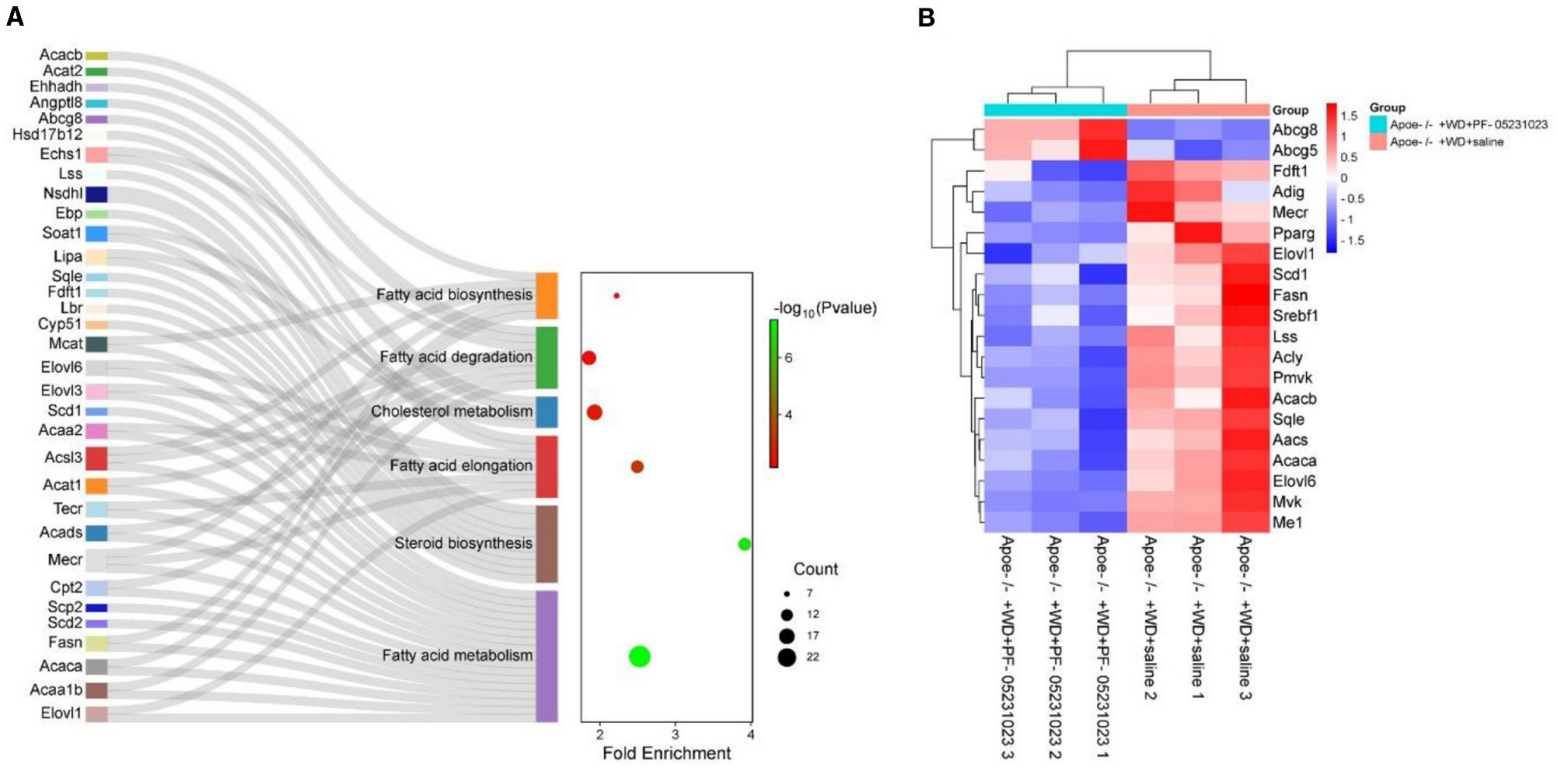
**(A)** Kyoto Encyclopedia of Genes and Genomes (KEGG) analysis of genes related to lipid metabolism and some significantly different genes. **(B)** Heat map depicting changes in candidate gene expression related to lipid metabolism. Blue indicates significant downregulation of gene expression (*p* < 0.05), whereas red indicates significant upregulation of genes (*p* < 0.05).

Six pathways were closely related to lipid metabolism in the KEGG analysis, including fatty acid metabolism, steroid biosynthesis, fatty acid elongation, fatty acid biosynthesis, fatty acid degradation, and cholesterol metabolism, as shown in Figure 9. In the fatty acid metabolism pathway, key genes involved in fatty acid synthesis, such as fatty acid synthase (*Fasn*), acetyl CoA carboxylase alpha (*Acaca*), and acetyl CoA carboxylase beta (*Acacb*), were significantly downregulated, whereas key genes involved in fatty acid oxidation, such as *Cpt1ab*, were upregulated. In the fatty acid elongation pathway, *Mecr*, very long chain fatty acid elongase 1 (*Elovl1*), and very long chain fatty acid elongase 6 (*Elovl6*) were significantly downregulated. In the steroid biosynthesis pathway, genes related to cholesterol synthesis, such as farnesyl-diphosphate farnesyltransferase 1 and squalene epoxidase, were significantly downregulated. In addition, steroyl CoA depletion 1 (*Scd1*) and adipogenin, which are involved in fat production, were significantly downregulated, whereas adenosine triphosphate-binding cassette transporter G8 (*Abcg8*), which promotes cholesterol efflux, was significantly upregulated. The levels of sterol regulatory element binding transcription factor 1 (*Srebf1*) also significantly decreased. Table 3 provides a detailed description of the genes involved in these pathways.

**Table 3:** DEGs involved in *de novo* adipogenesis, cholesterol biosynthesis, and fatty acid elongation pathways.

**Gene**	**Description**	**Log_2_FC**	***q*-Value**
*Aacs*	Acetoacetyl-CoA synthetase	−1.17186	0.000651
*Abcg8*	Adenosine triphosphate-binding cassette transporter G8	4.083074	0.011935
*Acaca*	Acetyl-CoA carboxylase alpha	−1.45815	9.53E−05
*Acacb*	Acetyl-CoA carboxylase beta	−1.09862	0.018698
*Acly*	Adenosine triphosphate citrate lyase	−1.83961	0.000663
*Adig*	Adipogenin	−2.0744	7.65E−06
*Elovl1*	Very long chain fatty acid elongase 1	−0.73961	0.004791
*Elovl6*	Very long chain fatty acids elongase 6	−2.31099	2.56E−07
*Fasn*	Fatty acid synthase	−1.8984	2.83E−05
*Fdft1*	Farnesyl-diphosphate farnesyltransferase 1	−1.11442	0.002743
*Lss*	Lanosterol synthase	−1.12141	0.01039
*Me1*	Malic enzyme 1	−1.93987	3.80E−07
*mecr*	Mitochondrial trans-2-enoyl-CoA reductase	−1.37633	1.43E−05
*Mvk*	Mevalonate kinase	−1.35399	2.01E−07
*Pmvk*	Phosphomevalonate kinase	−1.76177	2.03E−08
*Pparg*	Peroxisome proliferative activated receptor gamma	−1.23941	0.000471
*Scd1*	Stearoyl-CoA desaturase 1	−0.87024	0.004249
*Sqle*	Squalene epoxidase	−1.0713	0.002529
*Srebf1*	STEROL regulatory element binding transcription factor 1	−1.01262	0.010143

### 3.7 Real-time fluorescence quantitative PCR validation results

**Figure 10:**
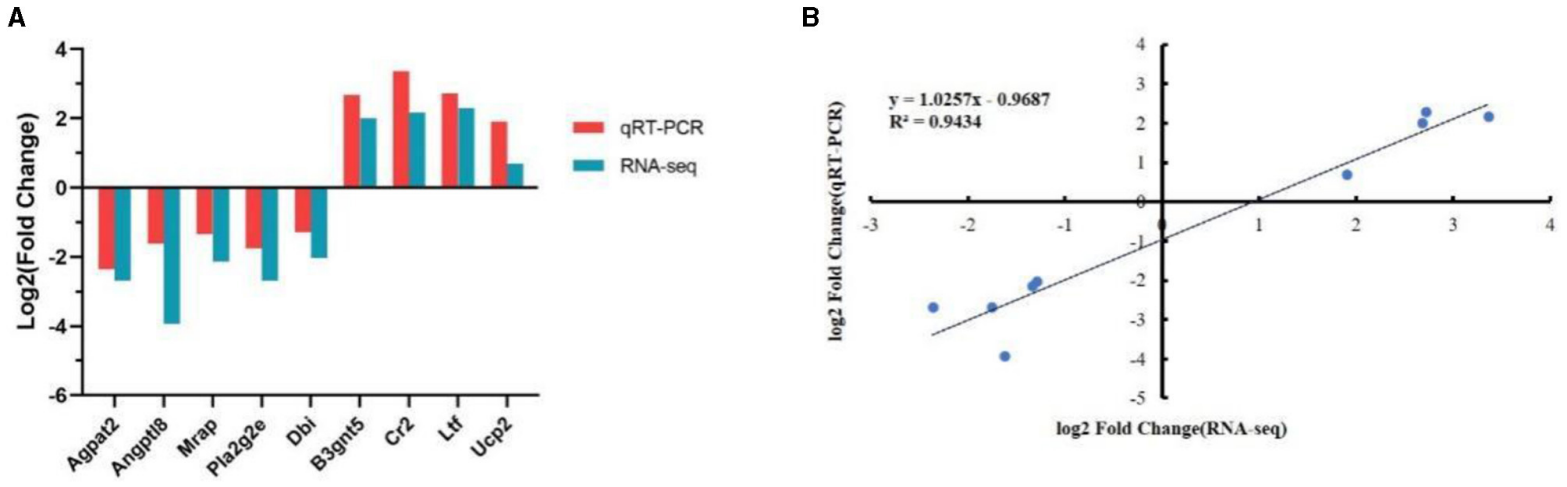
Validation of the selected differentially expressed genes (DEG) using quantitative reverse transcription PCR (qRT-PCR). **(A)** Comparison of expression trends between the RNA sequencing (RNA-Seq) and qRT-PCR data. **(B)** Correlation between selected gene expression ratios measured using RNA sequencing and qRT-PCR.

To verify the reliability of the transcriptional data, we randomly selected nine DEGs with high expression levels for quantitative PCR (qPCR) analysis. The qPCR results were consistent with the trends observed in the transcriptome analysis, confirming the accuracy and reliability of the transcriptome sequencing results (Figure 10A). Moreover, a high correlation was observed between the qPCR and RNA-Seq data, with *R*^2^ = 0.943 (Figure 10B).

## 4 Discussion

FGF21 analogs have been studied in clinical trials for type 2 diabetes and NAFLD and have demonstrated generally good tolerability. This study confirms that the long-acting FGF21 analog PF-05231023 reduces the accumulation of atherosclerotic plaques and improves lipid metabolism in the liver and adipose tissue of *Apoe*^−/−^ mice, thereby indicating the clinical potential of the FGF21 analog in treating AS. In this study, the body weights of *Apoe*^−/−^ mice treated with PF-05231023 gradually decreased until changes were observed prior to sampling. This result aligns with those of previous studies that reported weight loss in response to administration of FGF21 analogs in diet-induced obese mice (DIO) (22), and in Gubra Amylin NASH DIO mice (10). Although some clinical trials have suggested weight reduction after FGF21 analog treatment in subjects with obesity, type 2 diabetes, and fatty liver disease, the evidence for weight loss in subjects with obesity remains inconclusive (23). For example, in a clinical trial evaluating the safety and tolerability of PF-05231023 in patients with obesity with hypertriglyceridemia, PF-05231023 reduced TG and low-density lipoprotein cholesterol levels without causing body weight reduction (24).

After 7 weeks of treatment, the WT + Control and *Apoe*^−/−^ + Control groups showed minimal changes in body fat percentage. Conversely, both the PF-05231023 treatment and the physiological saline control groups showed a declining trend in body fat percentage, with the PF-05231023 treatment group demonstrating a more significant decrease. We hypothesize that this reduction in body fat percentage is likely attributed to the weekly intraperitoneal injections administered to both mouse groups, causing a certain degree of stress. This is supported by the observation that the two control groups, which did not receive intraperitoneal injections, did not show significant changes in body fat. FGF21 reduces blood sugar levels in diabetic rodents, obese mice, and monkey models (10). However, this study found that although there was no statistically significant difference in fasting blood glucose levels between *Apoe*^−/−^ mice injected with PF-05231023 and those injected with physiological saline, the former group showed an improvement in glucose tolerance. PF-05231023 does not significantly affect blood glucose control (11, 15). These minor differences in glucose improvement may be attributed to the slight differences in the pharmacology of FGF21 analogs (15). Additionally, injection of PF-05231023 had no significant effect on the reduction of plasma lipids in *Apoe*^−/−^ mice. Similarly, no significant reduction in plasma TG levels was detected in DIO mice treated with FGF21 (25). A study conducted on obese Göttingen mini pigs also found that FGF21 had no effect on pig plasma lipids, partly because of the very low circulating lipid levels in the mini pigs, which rendered FGF21 ineffective in reducing pig plasma lipids (26). The reason plasma lipid levels exhibited no significant effect in the current study remains unclear and could be a topic for further research. Numerous studies have investigated the role of FGF21 in AS. For example, a study using *Apoe*^−/−^ mice demonstrated that FGF21 alleviated AS by enhancing Fas-mediated apoptosis (27). Another study reported that FGF21 upregulates autophagy-mediated cholesterol efflux via receptor for activated C kinase 1 to inhibit AS (17). Furthermore, FGF21 alleviates AS by inhibiting focal pyroptosis in vascular endothelial cells, mediated by the NLR family pyrin domain containing three inflammasome (28). Thus, several mechanisms have been proposed for the inhibition of AS. In our study, PF-05231023 administration reduced fat accumulation in the liver and significantly reduced TC and TG content in the liver. This result aligns with the conclusion that PF-05231023 treatment reduces liver TC and TG in NAFLD, and reduces plasma liver injury markers alanine aminotransferase and aspartate aminotransferase (10). Additionally, our results are also consistent with research indicating that FGF21 improved lipid droplet size in the liver (16), as did the FGF21 analog LY2405319 (12). In various diet-induced, genetically modified, or chemically induced models, PF-05231023 treatment reduces the weight of brown fat, subcutaneous fat, and white adipose tissue, and it reduces adipocyte size in the white adipose tissue around the ovaries. FGF21-specific-knockout mice exhibit weight gain, decreased lipolysis, and reduced energy expenditure in the adipose tissue, leading to adipose tissue hypertrophy (11). However, FGF21 treatment reduces weight gain, improves lipid levels in adipocytes, and reduces inflammatory cell infiltration (29).

To better understand the molecular pathways associated with PF-05231023 and its effect on lipid metabolism in adipose tissue, we analyzed gene expression in the white adipose tissue around the ovaries. First, we annotated DEGs using the KEGG database and noted that most of the DEGs were concentrated in the categories related to metabolism and human diseases. By combining KEGG and GO functional analyses, we observed that pathways related to metabolism and human disease were downregulated in the differential gene set. Enrichment results for human disease show that PF-05231023 has a significant positive impact on the pathways associated with neurodegenerative diseases, including Alzheimer's disease (30), Parkinson's disease (31, 32), Huntington's disease, and amyotrophic lateral sclerosis. This finding suggests a potential role for long-acting FGF21 analogs in neuroprotection. FGF21 can modulates anti-inflammatory and antioxidant stress responses through nuclear factor kappa-B and adenosine 5′-monophosphate-activated protein kinase/protein kinase B signaling pathways, thereby protecting nerves from damage and mitigating neurodegenerative changes (33). Furthermore, our analysis revealed that PF-05231023 significantly downregulated the expression of genes related to NAFLD. This downregulation would lead to an improvement in the symptoms of this disease (34). This beneficial effect of PF-05231023 may be attributed to the direct regulation of lipid metabolism through insulin, resulting in reduced lipid accumulation in the liver. In the metabolic category, we identified several DEGs involved in metabolic pathways and lipid metabolism. We found that PF-05231023 significantly downregulated *Srebf1*, further leading to a decrease in the expression of *Fasn, Acaca, Acacb*, and *Scd1*. *Srebf1* is an important transcription factor that regulates lipid metabolism related enzymes primarily expressed in the liver and adipocytes. The transcription factor induces *de novo* adipogenesis by regulating the expression of adipogenic genes, such as *Acaca* and *Acacb, Fasn* (35), and *Scd1* (36, 37). *Acaca* and *Acacb* catalyze rate-limiting reactions in the biogenesis of long-chain fatty acids (38). *Fasn* is a key synthase in the process of fatty acid biosynthesis (38), which catalyzes *de novo* fatty acid synthesis by converting acetyl CoA and malonyl CoA to palmitic acid esters (39), and *Scd1* regulates the expression of unsaturated fatty acid biosynthesis genes and mitochondrial fatty acid oxidation (38). Moreover PF-05231023 also reduced the expression of adenosine triphosphate citrate lyase, which is an important enzyme in the cholesterol and fatty acid biosynthesis pathway upstream of *Fasn* (38), thereby promoting the biosynthesis of cholesterol and fatty acids by producing acetyl CoA from citrate (40). *Elovl1* and *Elovl6* are downregulated by *Srebf1*, which also leads to a decrease in fatty acid synthesis. Moreover, we observed a significant upregulation of *Abcg8* and an upregulation trend of adenosine triphosphate-binding cassette transporter G5 (*Abcg5*). The ABCG5/ABCG8 complex transports cholesterol and phytosterols from intestinal cells to the intestinal cavity for fecal treatment, thereby promoting cholesterol efflux and reducing cholesterol accumulation (41).

## 5 Conclusion

In summary, we confirmed that PF-05231023 reduces liver cholesterol and TG levels as well as lipid deposition in WD-fed *Apoe*^−/−^ mice and has the potential to counteract the development of AS. Further analysis of adipose tissue indicated that PF-05231023 reduces the expression of genes involved in adipogenesis, cholesterol synthesis, and fatty acid derivative pathways. Moreover, we found that the specific mechanism by which PF-05231023 inflicts these effects is to inhibit the expression of *Srebf1* and further inhibit the expression of genes related to lipid generation, thereby reducing lipid synthesis. However, the specific role of this pathway requires further investigation.

### 5.1 Study limitations

In the current study, we revealed the ameliorative effects of PF-05231023 on lipid metabolism as well as inhibitory effects on the expression of lipogenesis-related genes. However, the specific pathways regulated by PF-05231023 remain to be fully elucidated. Hence, the following aspects need to be further investigated: the mechanism by which PF-05231023 down-regulates *Srebf1* expression, whether *Srebf1* is linked to the expression of other lipogenesis-related genes, and the regulatory pathways between *Srebf1* and other lipogenesis-related genes.

## Data availability statement

The datasets presented in this study can be found in online repositories. The names of the repository/repositories and accession number(s) can be found below: NCBI SRA (accession: PRJNA1113307).

## Ethics statement

The procedures for animal handling for experiments were approved by the Committee of Experimental Animal Management at South China Agricultural University, China (protocol number: CEAM-SCAU-2023-0002). Moreover, all applicable rules and regulation of the organization and government were followed regarding the ethical use of experimental animals. The studies were conducted in accordance with the local legislation and institutional requirements. Written informed consent was obtained from the owners for the participation of their animals in this study.

## Author contributions

JZ: Conceptualization, Data curation, Formal analysis, Investigation, Methodology, Software, Supervision, Validation, Visualization, Writing – original draft, Writing – review & editing. XL: Investigation, Methodology, Validation, Visualization, Writing – review & editing. JY: Investigation, Methodology, Validation, Visualization, Writing – review & editing. SZ: Investigation, Methodology, Validation, Visualization, Writing – review & editing. LL: Formal analysis, Investigation, Methodology, Visualization, Writing – review & editing. HW: Conceptualization, Funding acquisition, Methodology, Project administration, Resources, Supervision, Writing – review & editing.

## References

[1] Ng M Fleming T Robinson M Thomson B Graetz N Margono C. Global, regional, and national prevalence of overweight and obesity in children and adults during 1980-2013: a systematic analysis for the Global Burden of Disease Study 2013 [published correction appears in Lancet. 2014 Aug 30;384(9945):746]. Lancet 384. (2014) 766–81. 10.1016/S0140-6736(14)60460-8 24880830 PMC4624264

[2] Samuel VT Shulman GI. The pathogenesis of insulin resistance: integrating signaling pathways and substrate flux. J Clin Invest. (2016) 126:12–22. 10.1172/JCI77812 26727229 PMC4701542

[3] Shulman GI. Ectopic fat in insulin resistance, dyslipidemia, and cardiometabolic disease [published correction appears in N Engl J Med. 2014 Dec 4;371:2241]. N Engl J Med. (2014) 371:1131–41. 10.1056/NEJMra1011035 25229917

[4] Dolegowska K Marchelek-Mysliwiec M Nowosiad-Magda M Slawinski M Dolegowska B. FGF19 subfamily members: FGF19 and FGF21. J Physiol Biochem. (2019) 75:229–40. 10.1007/s13105-019-00675-7 30927227 PMC6611749

[5] Fisher FM Maratos-Flier E. Understanding the physiology of fgf21. Annu Rev Physiol. (2016) 78:223–41. 10.1146/annurev-physiol-021115-105339 26654352

[6] Tan H Yue T Chen Z Wu W Xu S Weng J. Targeting FGF21 in cardiovascular and metabolic diseases: from mechanism to medicine. Int J Biol Sci. (2023) 19:66–88. 10.7150/ijbs.73936 36594101 PMC9760446

[7] Chen Z Yang L Liu Y Huang P Song H Zheng P. The potential function and clinical application of FGF21 in metabolic diseases. Front Pharmacol. (2022) 13:1089214. 10.3389/fphar.2022.1089214 36618930 PMC9810635

[8] Fisher FM Kleiner S Douris N Fox EC Mepani RJ Verdeguer F. FGF21 regulates PGC-1alpha and browning of white adipose tissues in adaptive thermogenesis. Genes Dev. (2012) 26:271–81. 10.1101/gad.177857.111 22302939 PMC3278894

[9] Gimeno RE Moller DE. FGF21-based pharmacotherapy – potential utility for metabolic disorders. TEM. (2014) 25:303–11. 10.1016/j.tem.2014.03.001 24709036

[10] Nielsen MH Gillum MP Vrang N Jelsing J Hansen HH Feigh M. Hepatoprotective effects of the long-acting fibroblast growth factor 21 analog pf-05231023 in the gan diet-induced obese and biopsy-confirmed mouse model of nonalcoholic steatohepatitis. Am J Physiol-Gastroint Liver Physiol. (2023) 324:G378–88. 10.1152/ajpgi.00157.2022 36852934

[11] Geng L Lam K Xu A. The therapeutic potential of FGF21 in metabolic diseases: from bench to clinic. Nat Rev Endocrinol. (2020) 16:654–67. 10.1038/s41574-020-0386-0 32764725

[12] Gaich G Chien JY Fu H Glass LC Deeg MA Holland WL. The effects of LY2405319, an FGF21 analog, in obese human subjects with type 2 diabetes. Cell Metab. (2013) 18:333–40. 10.1016/j.cmet.2013.08.005 24011069

[13] Huang J Ishino T Chen G Rolzin P Osothprarop TF Retting K. Development of a novel long-acting antidiabetic FGF21 mimetic by targeted conjugation to a scaffold antibody. J Pharmacol Exp Ther. (2013) 346:270–80. 10.1124/jpet.113.204420 23720456

[14] Veniant MM Komorowski R Chen P Stanislaus S Winters K Hager T. Long-acting FGF21 has enhanced efficacy in diet-induced obese mice and in obese rhesus monkeys. Endocrinology. (2012) 153:4192–203. 10.1210/en.2012-1211 22798348

[15] Talukdar S Zhou Y Li D Rossulek M Dong J Somayaji V. long-acting FGF21 molecule, PF-05231023, decreases body weight and improves lipid profile in non-human primates and type 2 diabetic subjects. Cell Metab. (2016) 23:427–40. 10.1016/j.cmet.2016.02.001 26959184

[16] Maeng HJ Lee GY Bae JH Lim S. Effect of fibroblast growth factor 21 on the development of atheromatous plaque and lipid metabolic profiles in an atherosclerosis-prone mouse model. Int J Mol Sci. (2020) 21:6836. 10.3390/ijms21186836 32957703 PMC7555741

[17] Xiaolong L Dongmin G Liu M Zuo W Huijun H Qiufen T. FGF21 induces autophagy-mediated cholesterol efflux to inhibit atherogenesis via rack1 up-regulation. J Cell Mol Med. (2020) 24:4992–5006. 10.1111/jcmm.15118 32227589 PMC7205825

[18] Lin Z Pan X Wu F Ye D Zhang Y Wang Y. Fibroblast growth factor 21 prevents atherosclerosis by suppression of hepatic sterol regulatory element-binding protein-2 and induction of adiponectin in mice. Circulation. (2015) 131:1861–71. 10.1161/CIRCULATIONAHA.115.015308 25794851 PMC4444420

[19] McGuinness OP Ayala JE Laughlin MR Wasserman DH. NIH experiment in centralized mouse phenotyping: the Vanderbilt experience and recommendations for evaluating glucose homeostasis in the mouse. Am J Physiol Endocrinol Metab. (2009) 297:E849–55. 10.1152/ajpendo.90996.2008 19638507 PMC2763792

[20] Pan L. Atlas of Experimental Pathology Techniques. Beijing: Science Press (2012).

[21] Centa M Ketelhuth DFJ Malin S Gisterå A. Quantification of atherosclerosis in mice. J Vis Exp. (2019) 148:e59828. 10.3791/59828 31259915

[22] Weng Y Chabot JR Bernardo B Yan Q Zhu Y Brenner MB. Pharmacokinetics (PK), pharmacodynamics (PD) and integrated PK/PD modeling of a novel long acting FGF21 clinical candidate PF-05231023 in diet-induced obese and leptin-deficient obese mice. PLoS ONE. (2015) 10:e0119104. 10.1371/journal.pone.0119104 25790234 PMC4366384

[23] Shao W Jin T. Hepatic hormone FGF21 and its analogues in clinical trials. Chronic Dis Transl Med. (2022) 8:19–25. 10.1016/j.cdtm.2021.08.005 35620160 PMC9126297

[24] Kim AM Somayaji VR Dong JQ Rolph TP Weng Y Chabot JR. Once-weekly administration of a long-acting fibroblast growth factor 21 analogue modulates lipids, bone turnover markers, blood pressure and body weight differently in obese people with hypertriglyceridaemia and in non-human primates. Diabetes Obes Metab. (2017) 19:1762–72. 10.1111/dom.13023 28573777

[25] Coskun T Bina HA Schneider MA Dunbar JD Hu CC Chen Y. Fibroblast growth factor 21 corrects obesity in mice. Endocrinology. (2008) 149:6018–27. 10.1210/en.2008-0816 18687777

[26] Christoffersen B Straarup EM Lykkegaard K Fels JJ Sass-Orum K Zhang X. FGF21 decreases food intake and body weight in obese gottingen minipigs. Diabetes Obes Metab. (2019) 21:592–600. 10.1111/dom.13560 30328263

[27] Yan X Gou Z Li Y Wang Y Zhu J Xu G. Fibroblast growth factor 21 inhibits atherosclerosis in apoe-/- mice by ameliorating fas-mediated apoptosis. Lipids Health Dis. (2018) 17:203. 10.1186/s12944-018-0846-x 30157856 PMC6114502

[28] Zeng Z Zheng Q Chen J Tan X Li Q Ding L. FGF21 mitigates atherosclerosis via inhibition of nlrp3 inflammasome-mediated vascular endothelial cells pyroptosis. Exp Cell Res. (2020) 393:112108. 10.1016/j.yexcr.2020.112108 32445748

[29] Xu J Lloyd DJ Hale C Stanislaus S Chen M Sivits G. Fibroblast growth factor 21 reverses hepatic steatosis, increases energy expenditure, and improves insulin sensitivity in diet-induced obese mice. Diabetes. (2009) 58:250–9. 10.2337/db08-0392 18840786 PMC2606881

[30] Chen S Chen ST Sun Y Xu Z Wang Y Yao SY. Fibroblast growth factor 21 ameliorates neurodegeneration in rat and cellular models of Alzheimer's disease. Redox Biol. (2019) 22:101133. 10.1016/j.redox.2019.101133 30785085 PMC6383137

[31] Desai BN Singhal G Watanabe M Stevanovic D Lundasen T Fisher FM. Fibroblast growth factor 21 (fgf21) is robustly induced by ethanol and has a protective role in ethanol associated liver injury. Mol Metab. (2017) 6:1395–406. 10.1016/j.molmet.2017.08.004 29107287 PMC5681240

[32] Yang C Wang W Deng P Li C Zhao L Gao H. Fibroblast growth factor 21 modulates microglial polarization that attenuates neurodegeneration in mice and cellular models of parkinson's disease. Front Aging Neurosci. (2021) 13:778527. 10.3389/fnagi.2021.778527 35002679 PMC8727910

[33] Kang K Xu P Wang M Chunyu J Sun X Ren G. Fgf21 attenuates neurodegeneration through modulating neuroinflammation and oxidant-stress. Biomed Pharmacother. (2020) 129:110439. 10.1016/j.biopha.2020.110439 32768941

[34] Raptis DD Mantzoros CS Polyzos SA. Fibroblast growth factor-21 as a potential therapeutic target of nonalcoholic fatty liver disease. Therap Clin Risk Manag. (2023) 19:77–96. 10.2147/TCRM.S352008 36713291 PMC9879042

[35] Palmer DG Rutter GA Tavare JM. Insulin-stimulated fatty acid synthase gene expression does not require increased sterol response element binding protein 1 transcription in primary adipocytes. Biochem Biophys Res Commun. (2002) 291:439–43. 10.1006/bbrc.2002.6467 11855808

[36] Foretz M Guichard C Ferré P Foufelle F. Sterol regulatory element binding protein-1c is a major mediator of insulin action on the hepatic expression of glucokinase and lipogenesis-related genes. PNAS. (1999) 96:12737–42. 10.1073/pnas.96.22.12737 10535992 PMC23076

[37] Yu S Song JH Kim HS Hong S Park SK Park SH. Patulin alleviates hepatic lipid accumulation by regulating lipogenesis and mitochondrial respiration. Life Sci. (2023) 326:121816. 10.1016/j.lfs.2023.121816 37271452

[38] Song T Wang P Li C Jia L Liang Q Cao Y. Salidroside simultaneously reduces de novo lipogenesis and cholesterol biosynthesis to attenuate atherosclerosis in mice. Biomed Pharmacother. (2021) 134:111137. 10.1016/j.biopha.2020.111137 33341055

[39] Su M Cao D Wang Z Duan Y Huang Y. Fatty acid synthase inhibitor platensimycin intervenes the development of nonalcoholic fatty liver disease in a mouse model. Biomedicines. (2022) 10:5. 10.3390/biomedicines10010005 35052685 PMC8773228

[40] Burke AC Huff MW. Atp-citrate lyase: genetics, molecular biology and therapeutic target for dyslipidemia. Curr Opin Lipidol. (2017) 28:193–200. 10.1097/MOL.0000000000000390 28059952

[41] Yu XH Qian K Jiang N Zheng XL Cayabyab FS Tang CK. ABCG5/ABCG8 in cholesterol excretion and atherosclerosis. Clin Chim Acta. (2014) 428:82–8. 10.1016/j.cca.2013.11.010 24252657

